# Blusher mushroom (*Amanita rubescens* Pers.): A Study of Mercury Content in Substrate and Mushroom Samples from Slovakia with Respect to Locality and Developmental Stages

**DOI:** 10.1007/s12011-024-04280-8

**Published:** 2024-06-28

**Authors:** Lenka Demková, Marek Šnirc, Ivona Jančo, Ľuboš Harangozo, Martin Hauptvogl, Lenka Bobuľská, Vladimír Kunca, Július Árvay

**Affiliations:** 1 Department of Ecology, Faculty of Humanities and Natural Sciences, University of Prešov, 17. Novembra 1, 081 16 Prešov, Slovak Republic; 2 Institute of Food Sciences, Faculty of Biotechnology and Food Sciences, Slovak University of Agriculture in Nitra, Tr. A. Hlinku 2, 949 76 Nitra, Slovak Republic; 3 AgroBioTech Research Centre, Slovak University of Agriculture in Nitra, Tr. A. Hlinku 2, 949 76 Nitra, Slovak Republic; 4 Department of Sustainable Development, Faculty of European Studies and Regional Development, Slovak University of Agriculture in Nitra, Tr. A. Hlinku 2, 949 76 Nitra, Slovak Republic; 5 Department of Applied Ecology, Faculty of Ecology and Environmental Sciences, Technical University in Zvolen, T. G. Masaryka 24, 960 01 Zvolen, Slovak Republic

**Keywords:** *Amanita rubescens*, Contamination factor, Health risk, Bioaccumulation factor, Target hazard quotient, Developmental stages

## Abstract

The edible mushroom *Amanita rubescens* Pers., regularly collected and consumed in Slovakia, was assessed for health risk due to the mercury content in its fruiting body parts. For this purpose, 364 both from the soil/substrate and mushroom samples from 40 localities in Slovakia were evaluated. At the same time, 21 samples of 7 developmental stages of the fruiting body of *A. rubescens* were taken in the Žakýlske pleso locality. The total mercury content in the soil and mushroom samples was determined using an AMA-254 analyzer. The contamination factor (*C*_f_) and index of geoaccumulation (*I*_geo_) were used to detect the level of soil pollution by mercury. The ability of *A. rubescens* to accumulate mercury from the soil environment was evaluated using the bioconcentration factor (*BCF*), and the distribution of mercury in the mushroom body was evaluated using the translocation quotient (*Q*_c/s_). To determine the health risks resulting from mushroom consumption, the percentages of provisional tolerable weekly intake (*%PTWI*) and target hazard quotient (*THQ*) were used. The obtained results have confirmed serious content of mercury soil pollution, especially in former mining areas, where the situation is alarming from a health risk point of view. Consumption of *A. rubescens* was found to be risky, not only in former mining areas, but higher values of mercury were also detected in other parts of Slovakia. Evaluation of the developmental stages of the fruiting body of *A. rubescens* showed that the highest bioconcentration factor was determined at developmental stage no. VI for caps with a value of 2.47 mg kg^−1^ and developmental stage VII for stipes with a value of 1.65 mg kg^−1^ DW.

**Supplementary Information:**

The online version contains supplementary material available at 10.1007/s12011-024-04280-8.

## Introduction

Fungi including mushrooms are crucial for the degradation, utilization, and transformation of organic and inorganic substrates [1, 2]. For centuries, edible mushrooms have been collected from forest areas or cultivated and consumed for their nutritional benefits, medicinal utility, and distinguishable flavor. Wild edible mushrooms are rich in many nutritionally beneficial components, such as amino acids, vitamins, polysaccharides, and lipids [3, 4]. However, mushrooms contain trace metals such as cadmium, mercury, lead, and silver, which are undesirable and toxic to mammals [5]. Owing to their nutritional, therapeutic, and economic benefits, mushrooms are currently consumed worldwide and are suitable food for vegetarians and vegans. In the last few decades, studies focusing on mushroom composition have become a matter of great interest to researchers because of their immunomodulatory, antimicrobial, antioxidant, anticancer, and antitumor properties [6–9]. Mushroom picking and eating are traditional activities in Slovakia, thanks to the lush forests and diverse types of mushrooms [10, 11]. For many years, Slovaks have been collecting mushrooms for both culinary and medicinal purposes, and this activity remains popular. *Boletus* spp., *Leccinum* spp., and *C. cibarius* are the most popular Slovak mushroom species [10]. Mercury is a global pollutant that has raised great concerns worldwide. Unlike many other risk metals, mercury can persist in the atmosphere for a long time and migrate long distances. Mercury is a carcinogenic metal toxin that considerably affects the immune system and causes blockage of the autonomic nervous system. Manifestations of mercury poisoning differ based on the chemical forms of mercury (organic or inorganic), the type of poisoning (acute or chronic), and the amount of mercury present.

High mercury concentrations can permanently damage the brain, kidneys, and fetal development [12, 13]. Inorganic mercury can be converted to highly neurotoxic methyl mercury (MeHg), which is bioaccumulated and biomagnified in the food chain, endangering human health. Mushrooms efficiently mobilize Hg from soil and its subsequent sequestration within fruiting bodies [8]. The differences in Hg uptake from soils among mushroom species may be genetically influenced. Edible parts of wild-growing mushrooms are products that show a higher content of Hg compared to fruits, vegetables, and other kinds of plant-based foods and animals [14–16].

The genus *Amanita* includes some of the best-known gourmet mushrooms, such as *A. rubescens* Pers*.*, *A. fulva*, *A. ovoidea*, *A. baccata*, *A. vaginata*, and *A. caesarea*; on the other hand, it is responsible for over 90% of lethal mushroom poisonings worldwide (*A. phalloides*) [17, 18]. Most of the species in this genus are ectomycorrhizal fungi that are associated with over ten tree families and play important roles in forest ecosystem health. Many species of the *Amanitaceae* family are inedible, for example, *A. pantherina*, *A. phalloides*, *A. virosa*, and *A. muscaria*, and are well-known for their psychedelic properties [9]. Although some of the listed mushrooms are edible and delicious, they are protected in Slovakia and, therefore, cannot be collected.

This publication is part of a series of articles devoted to the mercury content of the body parts of different mushroom species from various regions of Slovakia and the health risks resulting from their consumption [19–21]. *A. rubescens* is widely available and well-liked wild edible mushroom that exhibits excellent sensory quality. The fruiting bodies of *A. rubescens* can also bioaccumulate pollutants from the environment and ultimately pose a risk to consumers in the form of intoxication [14, 22–24].

In addition to geochemical anomalies, the primary source of soil pollution in Slovakia is a wide range of anthropogenic activities [25]. As mining activity has a long and rich history on the territory of Slovakia, it is one of the important polluters of the soil environment, along with industry, agriculture, and transport [26]. The threat in the form of increased content of toxic elements in the soil environment is mainly represented by former mining areas with untreated and un-reclaimed mining works.

The aims of this study were (a) to evaluate soil mercury pollution within Slovakia using the contamination factor (*C*_f_) and index of geoaccumulation (*I*_geo_); (b) to evaluate the relationship between soil mercury pollution and mercury content in mushroom body parts using the bioconcentration factor (*BCF*); (c) to determine the health risks resulting from the consumption of mushrooms using the percentage of provisional tolerable weekly intake *(%PTWI*) and the target hazard quotient (*THQ*); and (d) to evaluate the mercury content in the body of *A. rubescens* depending on the stage of development.

## Material and Methods

### Substrate and Mushroom Sampling and the Preparation Before Analysis

**Fig. 1:**
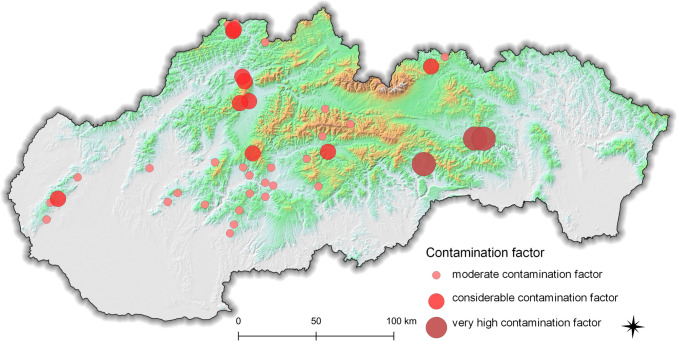
Average mercury contamination factor values (*C*_f(Hg)_) determined in 40 sampling localities within Slovakia

Samples of the edible mushroom *A. rubescens* (*n* = 364) were collected at 40 sampling localities within Slovakia from 2015 to 2021. The sampling localities are shown in Fig. 1S. The number of samples belonging to a particular locality ranged from 5 to 20. Simultaneously, the *A. rubescens* samples at seven developmental stages were collected at the locality of Žakýlske pleso. In total, 21 samples (three samples from each of the 7 developmental stages of the fruiting body) were sampled. The samples were collected on 1 day from an area of 2 × 2 m, where all seven developmental stages of *A. rubescens* were found. We chose such sampling conditions because of the probable presence of different generations of the same mycelium and at the same time low variability of the Hg content in the substrate. The mushroom samples were cleaned of larger impurities in situ and placed in ventilated polyethylene boxes for transportation. A corresponding soil/substrate sample (*n* = 364) of approximately 200 g from a depth of 0.10 m was taken at each sampling site.

Soil/substrate samples were taken as mixed samples from three random places in the vicinity up to 1 m from the mushroom sample and stored in re-sealable PE bags. Mushroom samples were processed under laboratory conditions on the same day as the sampling. The water content of the fresh samples, used for the analysis of the mercury transfer dynamics within individual developmental stages, was analyzed by moisture analyzer DLB 160-3A (Kern & Sohn GmbH, Germany). After washing in deionized water, the samples were divided into caps and stipes, cut into thin slices, and oven-dried with forced circulation (40 °C for ⁓24 h) in the laboratory oven (Memmert UF 110 m, Memmert & Co. KG, Schwabach, Germany). The dried samples were homogenized using a rotary homogenizer IKA A 10 basic (IKA-Werke GmbH & Co. KG, Staufen, Germany) and stored in re-sealable PE bags prior to the analysis. The soil/substrate samples were crushed, cleaned from impurities, and air-dried at room temperature for 3 weeks. Subsequently, the samples were sieved through a 2-mm sieve and stored in paper bags until analysis.

### Total Mercury Content Determination

Cold-vapor atomic absorption spectrometry (CV-AAS) using AMA-254 (AlTec spol. s.r.o., Prague, Czech Republic) coupled with an autosampler ASS-254 (AlTec spol. s.r.o., Prague, Czech Republic) was used for Hg content analysis in soil/substrate and mushroom samples. The detection and quantification limits were set at 0.0011 mg kg^−1^ and 0.0031 mg kg^−1^, respectively [27]. Quantitative determination of Hg was performed at *λ* = 253.7 nm. Two CRM materials were analyzed to check the quality and assurance of the measurements: ERM-CC 141, loam soil (IRMM Geel, Belgium) and ERM-CE 278 k, mussel tissue (IRMM Geel, Belgium). Each CRM was analyzed six times, and in each series, the blank three times. The recovery of the studied reference materials was as follows: ERM-CC 141, loam soil (98.6%; certified value, 0.083 mg kg^−1^ DW; determined value, 0.0818 mg kg^−1^ DW) and ERM-CE 278 k, mussel tissue (101.4%; certified value, 0.071 mg kg^−1^ DW; determined value, 0.072 mg kg^−1^ DW). The current water content was considered during measurement. Laboratory and analytical procedures focused on pre-treatment (drying, homogenization) have been described in detail in previous papers [19–21].

### Contamination Factor (*C*_f_) and the Index of Geoaccumulation (*I*_geo_)

To understand the ecological status of the soil environment in the former mining areas, the selected factors and indices were used. The contamination factor (*C*_f_) reflects the anthropogenic input of elemental pollution and is often used for soil evaluation worldwide [28]. It considers the content of risk elements from the surface of the soil and the background levels [29]. The contamination factor [30] is calculated as follows:

1 $${C}_{\text{f}}=\frac{{C}_{0-1}^{i}}{{C}_{n}^{i}}$$

where $C_{0-1}^i$ is the content of Hg measured in the soil/substrate sample and $C_n^i$ is the background level of Hg, which was for the Slovak soils set to 0.06 mg kg^−1^ [31]. The state of pollution was determined based on the following categories: low contamination factor (*C*_f_ < 1), moderate contamination factor (1 ≤ *C*_f_ < 3), considerable contamination factor (3 ≤ *C*_f_ < 6), very high contamination factor (*C*_f_ ≥ 6). 

The index of geoaccumulation (*I*_geo_) which was originally determined by Müller [32] is calculated as follows:

2 $${I}_{\text{geo}}=l{og}_{2}(Cn/1.5\times Bn)$$

where *C*_n_ is the measured concentration of the element in soil/substrate, and *B*_n_ is the geochemical background value of mercury in soils (0.06 mg kg^−1^) [31]. The values of *I*_*geo*_ are divided into seven categories [32]: background value (*I*_geo_ ≤ 0), uncontaminated (0 ≤ *I*_geo_ < 1), uncontaminated to slightly contaminated (1 ≤ *I*_geo_ < 2), slightly contaminated (2 ≤ *I*_geo_ < 3), moderately contaminated (3 ≤ *I*_geo_ < 4), strongly contaminated (4 ≤ *I*_geo_ < 5), a very strongly contaminated (*I*_geo_ ≥ 5).

#### Bioconcentration Factor (*BCF*) and Translocation Cap/Stipe Quotient (*Q*_c/s_)

Bioconcentration factor (*BCF*) was used to calculate the level of mercury accumulation from soil/substrate to the fruiting body of *A. rubescens* as follows:

3 $$BCF=\frac{{Hg}_{\text{ms}}}{{Hg}_{\text{ss}}}$$

where *Hg*_ms_ is the total content of mercury in mushroom samples (mg kg^−1^ DW) and *Hg*_ss_ is the total content of mercury in soil/substrate samples (mg kg^−1^ DW). The bioconcentration factor was set separately for caps and stipes of mushroom samples. *BCF* results indicate the excluder species (*BCF* < 1), indicator species (*BCF* = 1), and accumulators/hyperaccumulators (*BCF* > 1) [33].

The translocation cap/stipe quotient (*Q*_c/s_) was used to compare the level of Hg translocation within the fruiting body of *A. rubescens* mushroom. The translocation factor was calculated as follows [34]:

4 $${Q}_{\text{c}/\text{s}}=\frac{{Hg}_{\text{cap}}}{{Hg}_{\text{stipe}}}$$

where Hg_cap_ and Hg_stipe_ are the total content of mercury in caps and stipes, respectively.

### Health Risk Assessment

Considering that *A. rubescens* is one of the most frequently collected and consumed mushrooms in Slovakia, the percentage of provisional tolerable weekly intake *(%PTWI*) was used as an indicator of potential risk arising from long-term mushroom consumption. The *PTWI* for mercury was established as 0.004 mg kg^−1^ body weight per week [35]. Since we determined the average weight of an adult to be 70 kg, the provisional tolerable weekly intake was set to 0.28 mg per week. The percentage of *PTWI* was determined separately for caps and stipes, as follows:

5 $$\%PTWI=\frac{Hg\,in\,mushroom\times intake}{{PTWI}_{Hg}}\times100$$

 

where *Hg in mushrooms* is the total content of Hg in mg kg^−1^ determined in the mushroom sample (fresh weight). *Intake* is the estimated number of mushrooms consumed by an adult person. Based on the Statistical Office of the Slovak Republic [36], the amount of consumed “*other vegetables including mushrooms*” was 0.23 kg per week. The consumption of mushrooms from a locality may be considered a potential risk if the value exceeds 100%.

The targeted hazard quotient (*THQ*), which expresses the level of non-carcinogenic risk from the intake of pollutants, was used to determine the health risks resulting from the consumption of *A. rubescens* from different localities [37]. *THQ* is defined as the ratio of exposure to a toxic element and its highest reference dose, which has no adverse health effects on humans [38]. *THQ* can be calculated using the following equation:

6 $$THQ= \frac{\text{Efr }\times \text{ ED }\times \text{ ADC }\times {C}_{E}}{\text{RfDo }\times \text{ BW }\times \text{ ATn}}\times {10}^{-3}$$

where *Efr* is the exposure frequency (365 days); *ED* is the duration of the exposure (70 years); *ADC* is the average daily consumption of the mushrooms (33 g per day [35]); *CE* is the average Hg concentration in mushroom samples (mg kg^−1^ FW); *RfDo* is the oral reference dose for mercury (0.0003 mg kg^−1^ day^−1^) [39]; *BW* is the average adult body weight (70 kg); *ATn* is the average exposure time (25,550 days as the product of 365 days and 70 years); and 10^−3^ is used for the units conversion. If the *THQ* reaches a value lower than 1, it indicates a non-carcinogenic effect for the consumer. If the *THQ* reaches a value higher than 1, it is a sign of a higher increased threat in terms of carcinogenicity.

### Statistical Analysis and Map Preparation

All statistical analyses were performed using the PAST statistical program [40]. Data were tested for normality (Shapiro–Wilk test) and log + 1 was transformed before the analysis. Data are expressed using descriptive statistics as average ± standard deviation (minimum–maximum). Spearman’s correlation coefficient was used to determine the relationship between soil mercury content and its content in the body parts (caps and stipes) and the relationship between body parts in terms of total mercury content. The non-parametric Mann–Whitney *U* test was used to test for significant differences between caps and stipes in total mercury content, *BCF*, and *%PTWI*. The non-parametric Kruskal–Wallis test was used to determine the differences in *C*_f_ values between the evaluated localities. All the maps were created in the open-source QGIS software (version 3.26.2).

## Results and Discussion

The content of mercury determined in soil samples and mushroom (*A. rubescens*) body parts (caps and stipes) with the number of samples collected in individual locations is shown in Table 1.

**Table 1:** Mercury content (mg kg^−1^ DW) in substrate and mushroom samples from 40 sampling localities within Slovakia (average ± st. dev. (min–max))

Locality	Number of samples	Substrate	*A. rubescens*	
			Cap	Stipe
Badín	5	0.07 ± 0.01 (0.07–1.11)	0.25 ± 0.07 (0.13–0.55)	0.13 ± 0.04 (0.11–0.32)
Bojná	6	0.09 ± 0.01 (0.06–0.09)	0.86 ± 0.11 (0.58–0.94)	0.38 ± 0.05 (0.34–0.49)
Breziny	9	0.08 ± 0.02 (0.07–0.24)	0.26 ± 0.09 (0.03–0.74)	0.20 ± 0.04 (0.05–0.41)
Budča	7	0.09 ± 0.02 (0.08–0.15)	0.32 ± 0.03 (0.25–0.34)	0.19 ± 0.02 (0.16–0.24)
Drozdovo	9	0.12 ± 0.02 (0.05–0.24)	0.19 ± 0.05 (0.06–0.45)	0.13 ± 0.04 (0.03–0.41)
Hriňová	8	0.06 ± 0.01 (0.03–0.11)	0.27 ± 0.04 (0.10–0.49)	0.15 ± 0.02 (0.07–0.27)
Hronec	9	0.29 ± 0.03 (0.09–0.39)	0.36 ± 0.26 (0.22–2.36)	0.32 ± 0.25 (0.19–2.19)
Jabloňovce	13	0.05 ± 0.01 (0.03–0.13)	0.24 ± 0.07 (0.12–0.99)	0.13 ± 0.02 (0.07–0.31)
Klokočov	20	0.12 ± 0.16 (0.08–0.42)	0.47 ± 0.05 (0.25–1.01)	0.31 ± 0.06 (0.13–1.03)
Klubina	11	0.12 ± 0.01 (0.07–0.17)	0.45 ± 0.08 (0.13–1.09)	0.24 ± 0.11 (0.08–1.28)
Kostoľany pod Tribečom	16	0.08 ± 0.01 (0.05–0.19)	0.40 ± 0.05 (0.22–1.08)	0.24 ± 0.03 (0.13–0.61)
Krahule	6	0.26 + 0.01 (0.25–0.26)	0.56 ± 0.09 (0.46–0.65)	0.37 ± 0.06 (0.31–0.43)
Krompachy	6	2.38 ± 0.89 (1.54–5.32)	8.90 ± 1.03 (5.31–10.0)	4.33 ± 0.99 (2.75–6.89)
Krpáčovo	7	0.18 ± 0.03 (0.12–0.23)	0.18 ± 0.18 (0.09–0.86)	0.10 ± 0.13 (0.08–0.64)
Lehota pod Vtáčnikom	15	0.09 ± 0.01 (0.07–0.22)	0.22 ± 0.03 (0.08–0.49)	0.15 ± 0.03 (0.09–0.65)
Ľubietová	13	0.11 ± 0.01 (0.07–0.14)	0.16 ± 0.01 (0.09–0.25)	0.09 ± 0.01 (0.06–0.19)
Nižná Boca	6	0.15 ± 0.01 (0.12–0.18)	0.22 ± 0.03 (0.13–0.37)	0.18 ± 0.04 (0.08–0.35)
Nižnoslanská Baňa	8	3.01 ± 0.70 (2.56–8.40)	9.54 ± 1.69 (0.64–16.1)	5.89 ± 0.77 (0.43–7.65)
Olešná	8	0.30 ± 0.04 (0.14–0.44)	0.66 ± 0.10 (0.32–1.24)	0.51 ± 0.08 (0.19–0.88)
Pezinská Baba	5	0.11 ± 0.05 (0.08–0.30)	0.45 ± 0.13 (0.19–0.97)	0.25 ± 0.11 (0.15–0.75)
Pitelová	6	0.14 ± 0.05 (0.09–0.32)	0.17 ± 0.13 (0.14–0.69)	0.11 ± 0.10 (0.11–0.52)
Počúvadlianske jazero	6	0.09 ± 0.02 (0.03–0.14)	0.31 ± 0.05 (0.22–0.55)	0.21 ± 0.03 (0.14–0.33)
Raková	13	0.16 ± 0.02 (0.03–0.27)	0.90 ± 0.17 (0.19–2.52)	0.53 ± 0.56 (0.10–7.69)
Rosina	8	0.08 ± 0.09 (0.06–0.84)	0.40 ± 0.13 (0.16–1.33)	0.25 ± 0.09 (0.07–0.94)
Slaská	6	0.11 ± 0.02 (0.07–0.20)	0.36 ± 0.28 (0.15–1.80)	0.65 ± 0.16 (0.22–1.36)
Smolenice	12	0.07 ± 0.01 (0.05–0.12)	0.43 ± 0.03 (0.24–0.57)	0.12 ± 0.35 (0.24–0.02)
Staškov	12	0.28 ± 0.06 (0.10–0.64)	0.61 ± 0.31 (0.21–4.21)	0.45 ± 0.21 (0.10–2.54)
Stráňany	7	0.16 ± 0.01 (0.15–0.16)	0.42 ± 0.02 (0.36–0.43)	0.20 ± 0.01 (0.19–0.23
Svätý Jur	14	0.09 ± 0.01 (0.07–0.11)	0.10 ± 0.01 (0.08–0.14)	0.08 ± 0.01 (0.05–0.11)
Svätý Kríž	6	0.14 ± 0.05 (0.07–0.35)	0.31 ± 0.12 (0.17–0.81)	0.20 ± 0.07 (0.11–0.53)
Štefánska huta	11	4.80 ± 0.85 (1.28–10.0)	11.3 ± 1.27 (6.52–19.7)	6.12 ± 0.51 (4.04–9.32)
Trebostovo	8	0.35 ± 0.03 (0.28–0.40)	0.33 ± 0.04 (0.27–0.42)	0.21 ± 0.02 (0.19–0.26)
Valčianska dolina	6	0.13 ± 0.07 (0.08–0.48)	0.33 ± 0.78 (0.16–5.07)	0.22 ± 0.36 (0.11 + 0.39)
Veľká Lesná	8	0.29 ± 0.05 (0.19–0.58	1.14 ± 0.36 (0.17–2.95)	0.63 ± 0.23 (0.11–1.78)
Višňové	6	0.18 ± 0.03 (0.16–0.32)	0.66 ± 0.08 (0.29–0.79)	0.31 ± 0.03 (0.24–0.44)
Zlatno	10	0.08 ± 0.01 (0.04–0.17)	0.35 ± 0.04 (0.12–0.57)	0.23 ± 0.03 (0.03–0.37)
Zochova chata	20	0.23 ± 0.02 (0.06–0.43)	0.18 ± 0.05 (0.04–1.18)	0.09 ± 0.02 (0.04 + 0.41)
Zvolen	5	0.14 ± 0.01 (0.10–0.17)	0.94 ± 0.19 (0.27–1.25)	0.21 ± 0.17 (0.06–0.99)
Žakýlske pleso	7	0.10 ± 0.01 (0.09–0.15)	0.30 ± 0.02 (0.28–0.37)	0.19 ± 0.01 (0.17–0.23)
Žemberovce	6	0.12 ± 0.01 (0.06–0.15)	0.34 ± 0.12 (0.24–0.86)	0.21 ± 0.11 (0.15–0.73)

### Mercury Content in Soil/Substrate Samples

The average content of mercury in the soil was 0.12 ± 1.33 (0.03–10.0) mg kg^−1^ DW. The highest values were determined in former mining areas of Krompachy 2.38 ± 0.89 (1.54–5.32) mg kg^−1^ DW), Nižnoslanská Baňa 3.01 ± 0.70 (2.56–8.40) mg kg^−1^ DW, and Štefánská Huta 4.80 ± 0.89 (1.28–10.0) mg kg^−1^ DW. The localities reached significantly higher values of soil mercury content (*p* < 0.001) compared to others. From a statistical point of view, the soil mercury content determined in Štefanská Huta was also significantly higher than that of Krompachy and Nižnoslanská Baňa, while Krompachy and Nižnoslanská Baňa did not differ between themselves. According to Šefčík et al. [31], the average Hg content in Slovak soils was 0.06 mg kg^−1^ DW. Of the total number of evaluated sampling sites (*n* = 364), only 25 reached a value lower than the average for Slovakia. The limit value of mercury in the Slovak soils is set at 0.50 mg kg^−1^ based on the current legislation [41]. The evaluated soil samples exceeded the permissible limit in 30 cases, which was almost 8% of the sampling sites. In most cases, these have already been mentioned in former mining localities.

### Contamination Factor (*C*_f_) and Index of Geoaccumulation (*I*_geo_)

The contamination factor (*C*_f_) was used to evaluate the level of soil contamination and distinguish anthropogenic inputs from soil pollution [42]. The value of the mercury contamination factor was 2.07 ± 22.1 (0.46–167). According to the contamination factor values, 7.70% of the sampling sites had low contamination factor (*C*_f_ < 1), 58.8% were moderately contaminated (1 ≤ *C*_f_ < 3), 20.9% were considerably contaminated (3 ≤ *C*_f_ < 6), and 12.6% of the sampling sites were very highly contaminated by mercury (*C*_f_ ≥ 6). The average values of the contamination factors determined for each sampling locality are shown in Fig. 1. Based on the results obtained, we can conclude that mercury pollution in Slovak soils is serious. The highest values of contamination factor were found in former mining areas such as Štefanská Huta (max *C*_f_ = 167), Nižnoslanská Baňa (max *C*_f_ = 139), and Krompachy (max *C*_f_ = 88.6). These localities are known to be problematic in terms of pollution risk. Kimaková et al. [43], who evaluated the Hg content in arable soil in Slovakia, found that the Hg content in Štefanská Huta was 50 times higher than the maximum allowed limit level. Likewise, according to the long-term monitoring of the soil environment in the vicinity of Krompachy, the mercury content in the soil environment is extremely high and significantly exceeds the hygienic standards intended for healthy soils [44].

**Fig. 2:**
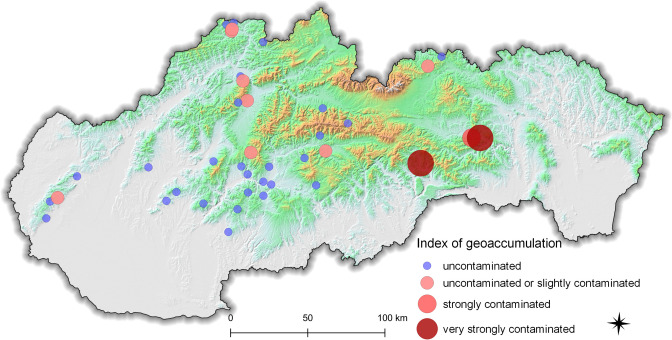
Average values of the index of geoaccumulation (*I*_geo_) determined in 40 sampling localities within Slovakia

The index of geoaccumulation (*I*_geo_) is useful for evaluating the anthropogenic contamination of soils by comparing soil concentrations with background concentrations [45] and gives us an understanding of the pollution status of the sampling sites. The average *I*_geo_ value was 0.46 ± 1.55 (it ranged between 1.69 and 6.79). Background values of mercury were determined in 31.1% of the samples, 35.4% were uncontaminated, 21.0% were uncontaminated or slightly contaminated, 5.20% were slightly contaminated, 0.60% were moderately contaminated, 1.50% were strongly contaminated, and 5.20% were very strongly contaminated by mercury. The average values for each sampling locality are shown in Fig. 2.

### The Mercury Content in *A. rubescens*

**Fig. 3:**
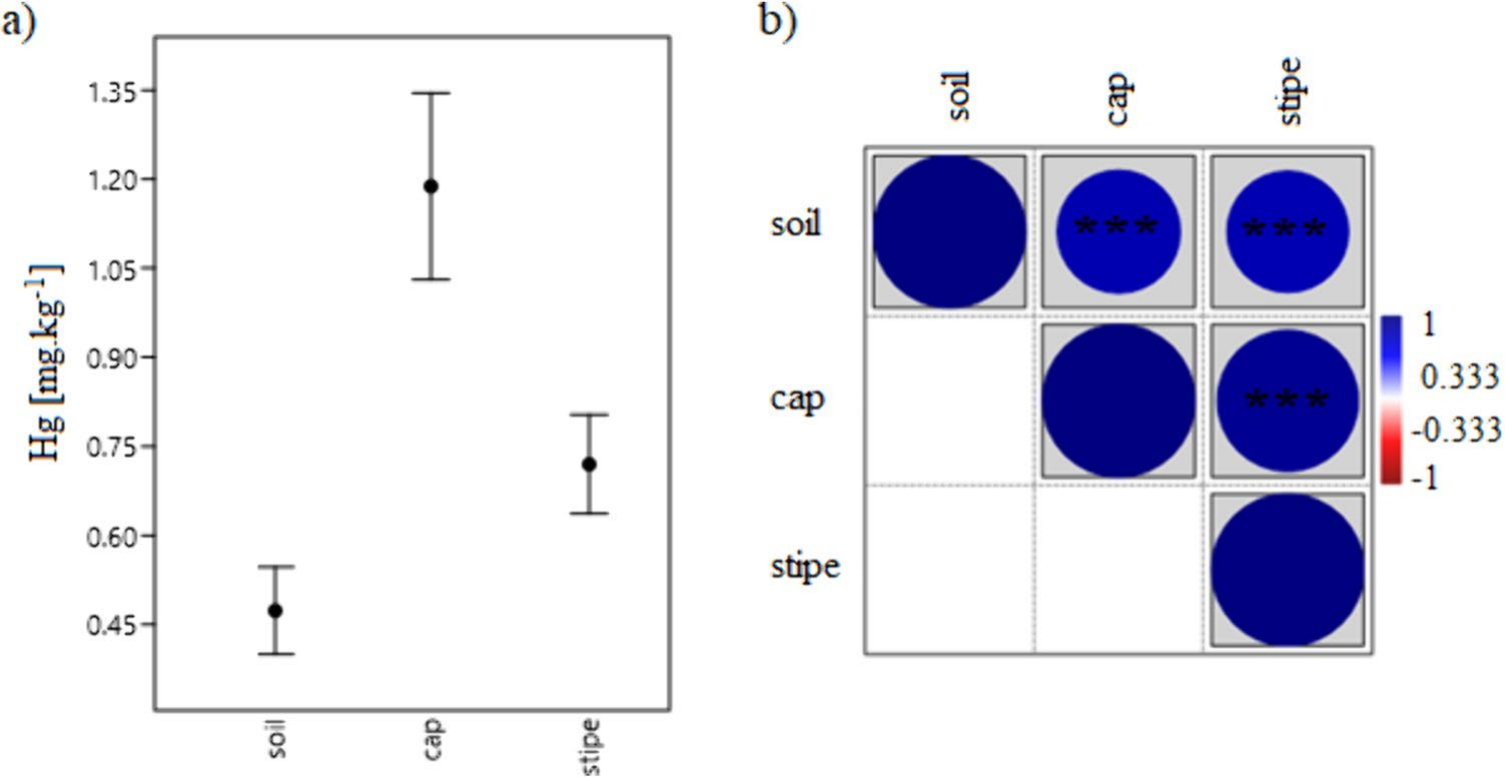
**a** Comparison of the mercury content in soil samples and mushroom body parts and **b** Spearman’s correlation relationship expressing the relationship between Hg content in soil and mushroom body parts (***p < 0.001)

The mercury content of mushrooms is influenced by the mercury content of the soil [46, 47]. The emerging bioindicative properties of mushrooms concerning the soil environment have been described in many studies [48, 49]. Several studies that focused on the impact of the environment on the pollution of the soil environment and subsequently on the growth of mushrooms have confirmed the significant impact of urbanization, transport, and other man-made activities [8, 50]. The authors also highlighted the ability of mushrooms to accumulate more toxic elements than other living organisms, especially plants [51–53]. Consistent with these findings, a significant positive correlation between the mercury content in *A. rubescens* body parts and the underlying soil samples (*p* < 0.001) was confirmed (Fig. 3). The ability of mushrooms to accumulate soil pollution was exceptional. Several studies have shown that this ability is species-specific [54] and influenced by the anatomy, physiology, and habitat conditions of the evaluated mushrooms [55]. In addition, the storage of risk elements in the bodies of mushrooms has certain specificities. The content of mercury in caps and stipes of *A. rubescens* was 0.37 ± 2.80 (0.03–19.7) mg kg^−1^ DW and 0.28 ± 1.50 (0.03–9.32) mg kg^−1^ DW, respectively. The content of mercury in caps of *A. rubescens* reached significantly higher values compared to stipes (*p* < 0.001). Our findings are consistent with those of Drewnowska et al. [9] who evaluated the content of Hg in caps and stipes of *A. rubescens* collected in Poland. In Central Europe, the risk element content of mushrooms was determined primarily in samples from environmentally polluted areas. In the Czech Republic, the values of mercury in *A. rubescens* sampled in the vicinity of the lead smelter were 12.0 ± 8.1 mg kg^−1^ DW, while control samples reached values of 1.3 ± 0.9 mg kg^−1^ DW [56]. In another Czech Republic historical silver-mining area, the values of mercury in *A. rubescens* were 1.55 ± 1.22 (0.25–4.0) mg kg^−1^ DW, while caps and stipes were not analyzed separately [57]. Demirbaş [58] who made research in the Black Sea region have found that the mercury content in *A. rubescens* was 0.42 ± 0.08 mg kg^−1^ DW.

### Bioconcentration Factor (*BCF*) and Translocation Quotient Cap/Stipe (*Q*_c/s_)

**Fig. 4:**
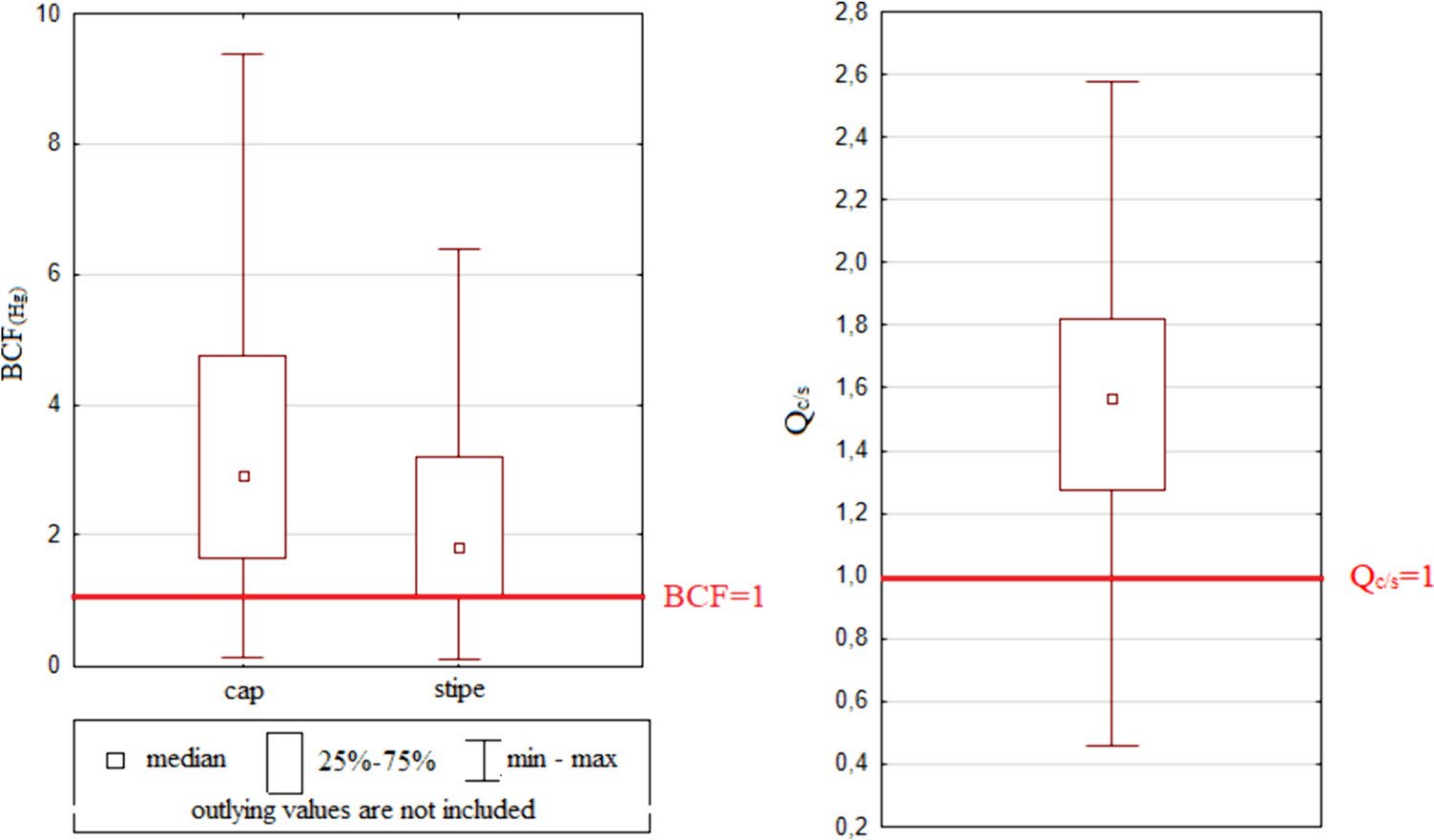
Bioconcentration factor (*BCF*) values and translocation quotient (*Q*_c/s_) values determined in *A. rubescens* mushroom samples

The bioconcentration factor was used to detect the ability of *A. rubescens* to accumulate mercury from the soil/substrate. The determined values differ significantly (*p* < 0.001) between caps 2.92 ± 3.57 (0.13–32.9) and stipes 1.80 ± 2.92 (0.08–31.9). These results are in accordance with the findings of Chudzyński and Falandysz [59], who claimed that the mercury content in caps is usually higher because of the higher metabolic activity and the higher content of proteins and enzymes (compared to stipes) that can bind Hg. Only 13.0% of the cap samples and 24.0% of the stipe samples reached BCF values lower than 1 (Fig. 4). Based on the results obtained, we can conclude that *A. rubescens* can be considered a mercury accumulator. The ability of mushrooms to accumulate mercury from the soil environment, and thus the values of the *BCF*, is also influenced by the type of substrate, climate, and agricultural or farming activities [60–62]. Andráš et al. [63] who evaluated the bioconcentration abilities of 13 mushroom species sampled in a former mining area (Slovakia) have found that only 2 species can be considered excluders (*BCF* < 1).

**Fig. 5:**
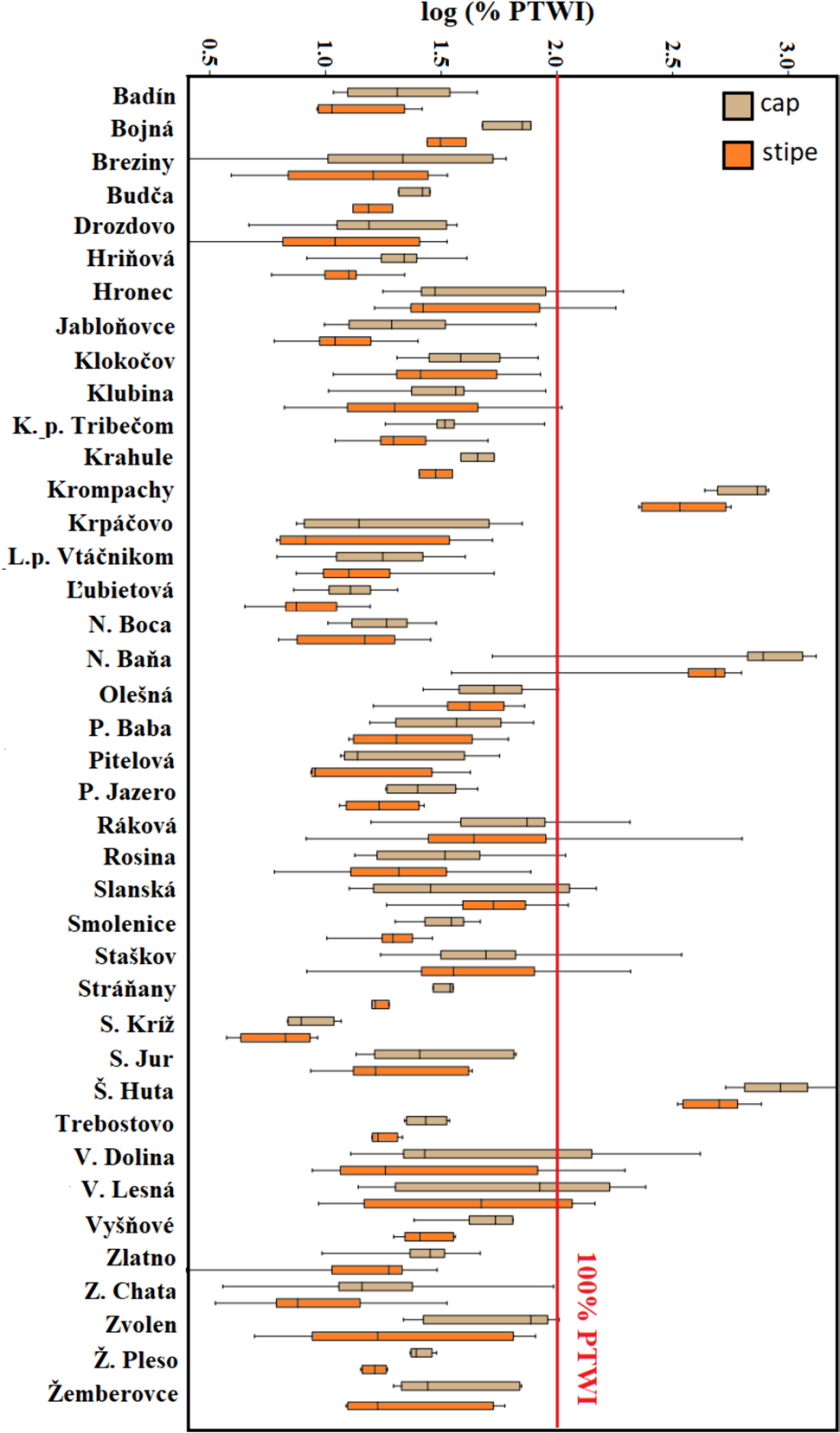
Percentage of provisional tolerably weekly intake values (%*PTWI*) determined in *A. rubescens* mushroom samples from the 40 sampling localities

The translocation quotient (*Q*_c/s_) was used to express the mobility of Hg in the fruiting bodies of mushrooms. The *Q*_c/s_ values determined for *A. rubescens* were 1.56 ± 2.51 (0.13–41.6) (Fig. 5). Values of *Q*_c/s_ higher than 1 indicate that the caps of the mushroom accumulate higher amounts of mercury than the stipes. The *Q*_c/s_ value was lower than one in only 9.0% of the evaluated samples. Higher concentrations of Hg are usually found in caps than in stipes; additionally, they are largely concentrated in hymenophore gills and tubes [64–66]. Likewise, our results showed higher values of Hg in caps, which are more often processed and consumed than stipes.

### Percentage of Provisional Tolerably Weekly Intake (*%PTWI*)

The higher content of mercury in the consumed products can cause serious health problems, which is why the Food and Agriculture Organization/World Health Organization [67] set safe levels of some risky elements in terms of provisional tolerable daily intake. If the level of 100% *PTWI* is exceeded, there is a real threat to consumer health. The results obtained showed that the *%PTWI* for caps 30.4 ± 232 (2.54–1617) reached significantly higher values (*p* < 0.01) compared to stipes 19.5 ± 123 (2.49–765). This level was significantly influenced by the locality of *A. rubescens* (Fig. 5). The level of 100% *PTWI* was exceeded in almost 12.0% of the cap samples and 10.0% of the stipe samples. The highest values were determined in former mining areas, which are considered problematic in terms of environmental quality for a long period [68, 69]. All of them (Krompachy, Štefanská Huta, and Nižnoslanská Baňa) are among the environmentally burdened territories and are registered in the Identification System of Environmental Burdens of the Slovak Republic [70]. As stated by several researchers, the consumption of mushrooms collected in the vicinity of mining areas always brings increased health risks [57, 71–73]. The values exceeding 100% of *PTWI* were also recorded at several sampling sites of Veľká Lesná locality which is part of the Pieniny National Park. The pollution in this area may be related to the proximity of large urban centers localized close to the northern border of Slovakia (in Poland) and the transmission of pollutants to this area by wet or dry deposition [74]. However, several studies have stated that the highest content of risk elements in the ambient air of High Tatras may be primarily caused by the proximity of mines and ore processing plants on the Polish side of the Tatras [75]. In addition to metal ore mining and metallurgy, some other industrial and economic activities also took place in the Polish Tatras such as quarries, sawmills, lime works, and two paper mills [76, 77].

### Target Hazard Quotient (THQ)

**Fig. 6:**
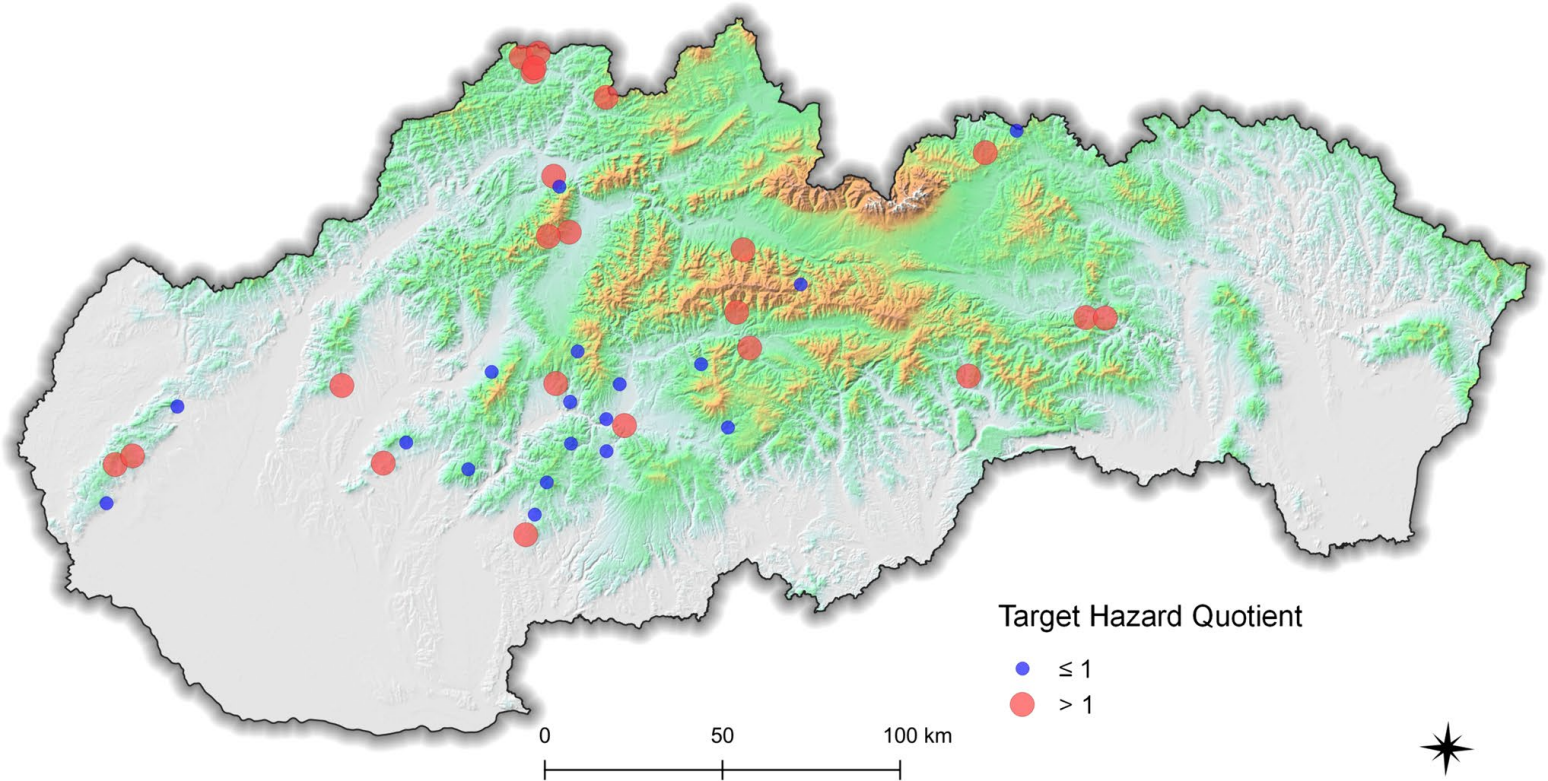
The THQ values determined in the mushroom samples in 40 sampling localities (red mark, at least one sample from the locality reached *THQ* > 1; blue mark, no sample reached *THQ* > 1)

The target hazard quotient was established to determine the health risks resulting from the long-term consumption of mushrooms, considering the highest safety reference dose of mercury. Food is considered safe if the value of 1 is not exceeded. The *THQ* values were determined for caps 0.58 ± 4.46 (0.05–31.0) and stipes 0.37 ± 2.36 (0.05–14.7) of *A. rubescens.* Value 1 was exceeded in 25% of cap samples and 17% of stipe samples. By comparing the locations where at least one sample was found to be risky (exceeding the *THQ* > 1 value), we found that out of the total number of 40 evaluated locations, a health risk related to the consumption of *A. rubescens* was detected in 22 (Fig. 6). This represents a much higher percentage (55%) of potentially risky locations compared to the *%PTWI*. It is due to the fact that *THQ* takes into consideration a health risk resulting from long-term exposure whereas *%PTWI* determines the acute exposure. All the locations determined as potentially risky by the *%PTWI* correlated with the *THQ* results.

### Mercury Bioconcentration Based on the Developmental Stages

**Fig. 7:**
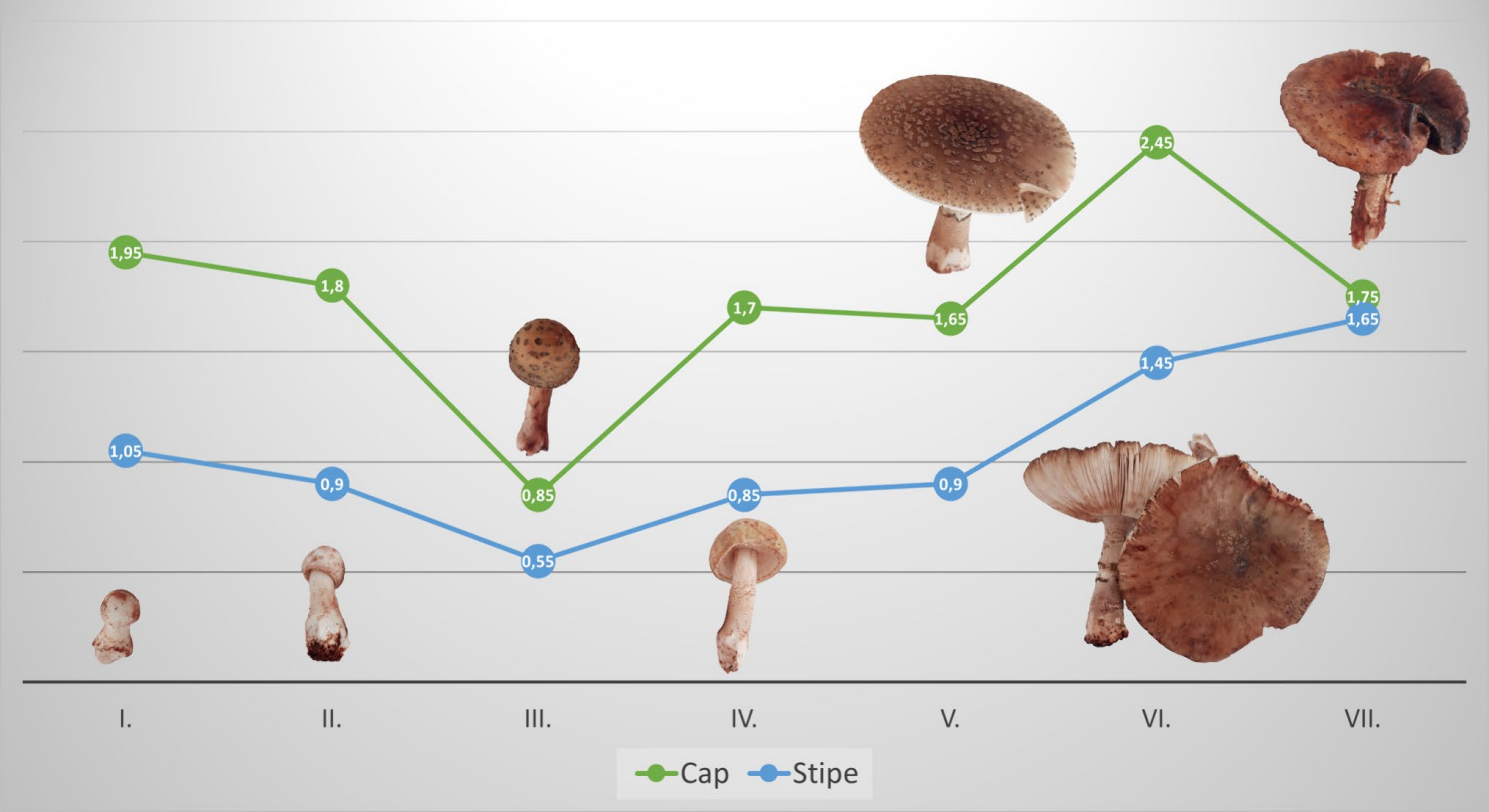
Average values of *BCF* determined in caps and stipes of *A. rubescens* mushroom with developmental stage visualization and age determination (I, < 1 day; II, 1–2 day(s); III, 2–3 days; IV, 3–4 days; V, 4–5 days; VI, 5–7 days; VII, > 7 days)

The mercury content in the caps and stipes of *A. rubescens* at different developmental stages is shown in Fig. 7. It has been found in earlier studies that the Hg content in the edible mushroom bodies is significantly influenced (besides the family, genus, and species affiliation) by developmental stage [78, 79]. The differences in Hg content during the developmental stages could be the result of the dilution effect when the content of the water in the cap changes. Additionally, the older mushroom bodies lose the vitality and the ability to transport nutrients, because the fruiting body of the fungus is dispersed, and its life cycle ends. The ability of *A. rubescens* to accumulate Hg from the soil in different developmental stages was also expressed by* BCF* values, separately for caps and stipes (Fig. 7). The results largely replicated the differences found in the total mercury content in individual developmental stages. In both cases (the total Hg content and *BCF*), it was confirmed that time also plays a key role, since longer exposure to mercury in the soil environment (mushrooms in older developmental stages) resulted in higher *BCF* values.

## Conclusion

The highest concentration of Hg in soil and *A. rubescen*s body samples was recorded in former mining areas. However, other areas of Slovakia cannot be considered uncontaminated. The high Hg content in the soil environment was also manifested by its increased concentrations in the edible mushroom *A. rubescens*, which can be considered an accumulator of mercury. More than half of the studied 40 locations can be considered risky when it comes to the consumption of *A. rubescens.* We found the highest health risks at the former mining localities, where the quality of the environment has been a known and unresolved problem for a long time. Considering the Hg content at different developmental stages of the *A. rubescens* body, we can conclude that from a health point of view, younger mushrooms are more suitable for human consumption.

## Supplementary Information

Below is the link to the electronic supplementary material.

Supplementary file1 (DOCX 964 KB)

## Data Availability

All data generated or analyzed during this study are included in this published article (and its supplementary information file).
