# Supplementary material for: Blusher mushroom (Amanita rubescens Pers.): A Study of Mercury Content in Substrate and Mushroom Samples from Slovakia with Respect to Locality and Developmental Stages
Source: Biol Trace Elem Res. 2024 Jun 28;203(3):1721–33. doi: 10.1007/s12011-024-04280-8 (PMC11872988; doi:10.1007/s12011-024-04280-8)
Supplement: Supplementary file 1 — Supplementary file1 (DOCX 964 KB) [file 12011_2024_4280_MOESM1_ESM.docx]

**Supplementary material**


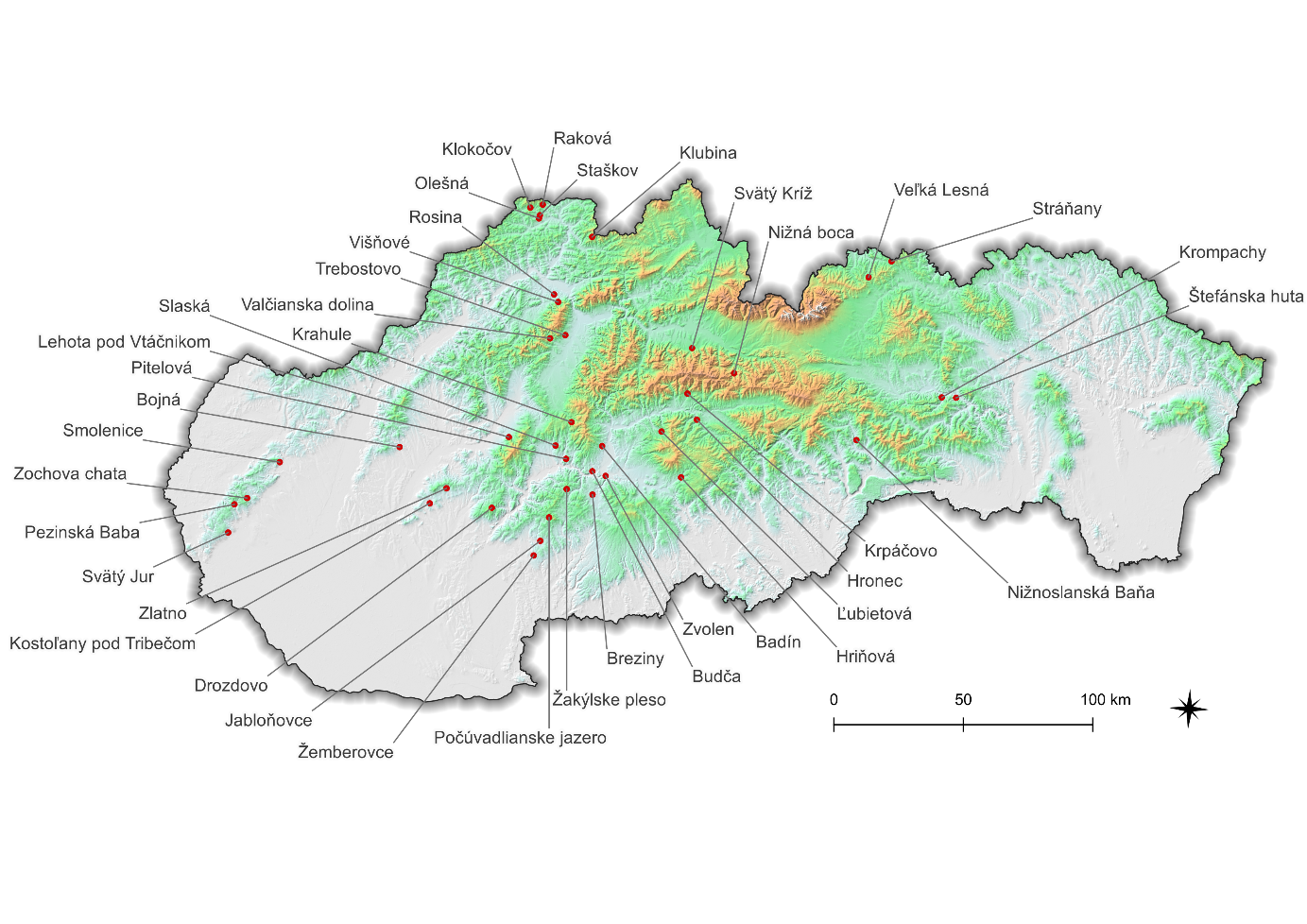


**Figure 1 S** Soil/substrate and mushroom sampling localities within Slovakia.
